# Advance telephone notification of follow-up in the healthlines study: a nested study of patients with depression

**DOI:** 10.1186/1745-6215-16-S2-P111

**Published:** 2015-11-16

**Authors:** Louisa Edwards, Chris Salisbury, Katy Garner, Kim Horspool, Alexis Foster, Alan Montgomery

**Affiliations:** 1 University of Bristol, Bristol, Bristol, UK; 2 University of Sheffield, Sheffield, UK; 3 University of Nottingham, Nottingham, UK

## Background

Advance notification may increase response to follow-up questionnaires among trial participants, but the benefit of this resource-intensive activity remains uncertain. This study investigated the effectiveness of pre-calling participants with depression ahead of follow-up in The Healthlines Study, a trial of a telehealth intervention.

## Methods

Participants in one of the three study centres were alternately allocated to either be telephoned 2-7 days before sending an 8-month follow-up questionnaire by post or email, or to be sent the questionnaire without pre-calling. All participants received up to five questionnaire completion reminders. The primary outcome was completion of the PHQ-9 outcome. Secondary outcomes were number of reminders and time to questionnaire completion.

## Results

201 participants were included in the nested study (100 to pre-calling), of a total of 609 in the Healthlines depression trial. Outcome completion was ≥90% in both arms (OR 0.82, 95% CI 0.31 to 2.19). Participants in the pre-calling arm were less likely to require a reminder (47% vs 62%, OR 0.46, 0.25 to 0.84), required fewer reminders (difference in means -0.6, -1.1 to -0.2), and completed follow-up quicker (median 8 vs 15 days, HR 1.24, 0.92 to 1.66) than participants who received no pre-calling.

## Conclusion

Outcome completion rate at 8-months follow-up was high. There was no evidence that pre-calling participants achieved better completion rates, nor reduced overall net effort required to achieve follow-up after taking into account pre-contacting these participants. Pre-calling may be helpful when timing of outcome completion is important.

